# A Comparison between BCI Simulation and Neurofeedback for Forward/Backward Navigation in Virtual Reality

**DOI:** 10.1155/2019/2503431

**Published:** 2019-10-09

**Authors:** Bilal Alchalabi, Jocelyn Faubert

**Affiliations:** Biomedical Engineering Department, University of Montreal, Montreal, Canada

## Abstract

A brain-computer interface (BCI) decodes the brain signals representing a desire to do something and transforms those signals into a control command. However, only a limited number of mental tasks have been previously investigated and classified. This study aimed to investigate two motor imagery (MI) commands, moving forward and moving backward, using a small number of EEG channels, to be used in a neurofeedback context. This study also aimed to simulate a BCI and investigate the offline classification between MI movements in forward and backward directions, using different features and classification methods. Ten healthy people participated in a two-session (48 min each) experiment. This experiment investigated neurofeedback of navigation in a virtual tunnel. Each session consisted of 320 trials where subjects were asked to imagine themselves moving in the tunnel in a forward or backward motion after a randomly presented (forward versus backward) command on the screen. Three electrodes were mounted bilaterally over the motor cortex. Trials were conducted with feedback. Data from session 1 were analyzed offline to train classifiers and to calculate thresholds for both tasks. These thresholds were used to form control signals that were later used online in session 2 in neurofeedback training to trigger the virtual tunnel to move in the direction requested by the user's brain signals. After 96 min of training, the online band-power neurofeedback training achieved an average classification of 76%, while the offline BCI simulation using power spectral density asymmetrical ratio and AR-modeled band power as features, and using LDA and SVM as classifiers, achieved an average classification of 80%.

## 1. Introduction

A BCI is a communication system that bypasses the body's neuromuscular pathways, measures brain activity associated with the user's intent, and translates it into corresponding control signals to an electronic device, only by means of voluntary variations of brain activity. Such a system appears as a particularly promising communication channel for persons suffering from severe paralysis, like those with the “locked-in” syndrome, and, as such, is locked into their own body without any residual muscle control [1, 2].

Studies to date show that humans can learn to use electroencephalographic (EEG) activity to control the movements of a cursor [3, 4, 5, 6] in one or two dimensions, or to control other devices such as spellers [7, 8]. Both actual movement and movement imagery are accompanied by changes in the amplitudes of certain EEG rhythms, specifically 8–12 Hz mu rhythms and 18–30 Hz beta rhythms [9, 10]. These power changes, named “event-related synchronization/desynchronization (ERS/ERD)” [10], are focused over the sensorimotor cortex in a manner consistent with the homuncular organization of this cortical region [2].

In the 1980s, Krusienski et al. started on EEG-based cursor control in normal adults using band power centered at 9 Hz. They used an autoregressive model to compute power in a specific frequency band, where the sum power was used in a linear function to control the cursor's direction of movements [11]. Nowadays, the world of the BCI is expanding very rapidly. One new field involves BCIs to control virtual reality (VR), including BCIs for games [12, 13, 14, 15] and for computer applications such as a virtual keyboard [16]. The virtual environment (VE) can provide an excellent testing ground for procedures that could be adapted to real-world scenarios, especially for patients with disabilities. If people can learn to control their movements or perform specific tasks in a VE, this could justify the much greater expense of building physical devices such as a wheelchair or robot arm that is controlled by a BCI. One of the main goals of implementing the BCI in VR is to understand how humans process dynamic visual scenes and how well they can interact with these natural environments.

The first efforts to combine VR and BCI technologies were in years 2000 and 2003 by Bayliss and Ballard who introduced a VR smart home in which users could control different things using a P300 BCI [17, 18].

Then in 2003, researchers showed that immersive feedback based on a computer game can help people learn to control a BCI based on imaginary movement more quickly than mundane feedback [19]. Researchers in [20] used the BCI for walking in a virtual street in 2005, for visiting and navigating in a virtual reality representation of the Austrian National Library in 2007 [21], and for exploring a smart virtual apartment in 2010 [22, 23].

In [24], researchers used the BCI to navigate in virtual reality with only beta waves, where a 35-year-old tetraplegic male subject learned to control a BCI, where the midcentral focused beta oscillations with a dominant frequency of approximately 17 Hz allowed the BCI to control the VE. Only one single EEG channel was recorded bipolarly at Cz (foot representation area). One single logarithmic band-power feature was estimated from the ongoing EEG. A simple threshold (TH) was used to distinguish between foot movement imagination (IC) and rest (INC). This study, which was based only on beta waves, has classified two different mental states: one directional movement (forward) and a rest state but not for backward movement.

Brosseau-Lachine et al. in [25, 26] psychophysically studied infants and made electrophysiological recordings of brain cells in cats' response to radial optic flow fields and found superior sensitivity to expansion versus contraction direction of motion in both studies. This is further supported by an imaging study with adults where the researchers have found a bias for expanding motion stimuli [27]. This dissociation may suggest that sensitivity to direction corresponding to forward locomotion (expansion) develops at a faster rate than that to opposite direction encountered when moving backward (contraction) [28].

Researchers in [27] found with PET scan that several loci of activation were observed for contraction and expansion conditions in the same areas of the human brain, but the increase in rCBF in contraction was much lower than that in the expansion condition in the right brain. Since researchers found that imagining a movement activates the brain the same way as watching or doing that movement does, then MI of navigation in a virtual reality scene with a strong optic flow feedback might activate the brain the same way the real navigation would do.

There is ample physiological and behavioral evidence that perceiving forward and backward movements are quite different in nature and therefore cannot be considered equivalent. Given that one of the BCI system's aims is developing brain-controlled movements, it is therefore important to address the distinction between efficiency of BCI-driven movements for these two critical direction controls.

So, we wanted to see in the present study if we could classify those two-directional movements in virtual reality from only 1, 2, or 3 EEG channels.

Therefore, the main goal of the present project was to enhance navigation in virtual reality with the brainwaves by using beta ERS obtained from a small number of channels. Furthermore, we wanted to see if we could classify two-directional movements (forward or backward) in virtual reality from these channels so that in the future, a subject could efficiently navigate by altering the brain waves and freeing the limbs for other activities.

## 2. Methodologies

### 2.1. Equipment

#### 2.1.1. Virtual Reality Equipment

**Figure 1:**
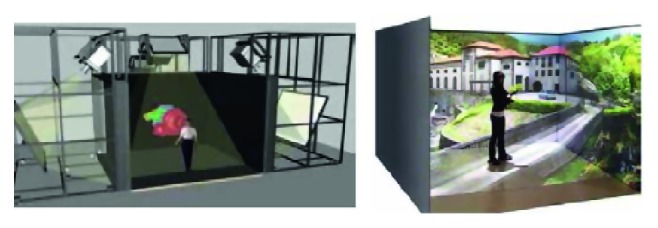
Icube.

To generate virtual environments, we used The EON Icube™, a PC-based multisided immersive environment in which participants are surrounded by virtual imagery and 3D sound (Figure 1).

Stimuli were generated on four synchronized HP Z800 workstations and were rear-projected onto four Da-Lite Fast-Fold 10′ wide ×7.5′ length/long wall screens (one frontal, two lateral, and one ground), from a distance of 4.10 m. The image was projected on the screens using 4 InFocus LP725 projectors that scan at 75 Hz with a resolution of 3,072 × 768,2500 ANSI lumen brightness and 2400 : 1 contrast ratio. Stereoscopic active lightweight shutter glasses and 12-DOF electromagnetic camera position trackers were also installed, and the result was complete immersion of the user in a virtual world [28].

#### 2.1.2. Virtual Reality Tunnel

As a virtual environment, we had chosen 3D Tunnel that can move in the anterior-posterior direction. The virtual tunnel respected all the aspects of a real physical tunnel (i.e., stereoscopy and size increase with proximity).

**Figure 2:**
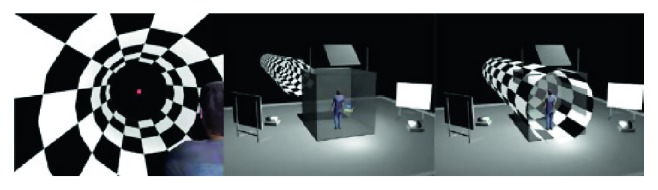
Virtual reality tunnel.

To program the tunnel, C++, OpenGL, and VR Juggler were used. The tunnel, depicted in Figure 2, was 3 m in diameter and 91 m long. Its front extremity was closed with a wall (subtending 2°) to reduce aliasing. Its back extremity was virtually located 7 m behind the subject [28]. A red fixation point (subtending 0.2°) was placed at the end of the tunnel at equal distance from the lateral wall.

Stimuli were either static or dynamic moving at a speed of 1.1 m/s. This velocity was perceived corresponding to normal gait. Proximal parts of the tunnel remained, while central parts were truncated and replaced by a black uniform field.

The texture was a pattern of alternating black and white squares. The association of shape (cylinder), texture, and perspective provided a radial flow to the central visual field and a lamellar flow to the peripheral visual field. This optic flow structure is the one for which the visual system is very sensitive and consequently quite responsive with respect to the control of stance [28]. The squares were all of the same size in the virtual world (corresponding to real-world conditions) but appeared smaller at distance due to perspective.

Consequently, sensitivity of the visual system for spatial frequencies and cortical magnification were essentially accounted for by this naturalistic stimulation [28].

#### 2.1.3. EEG Recordings

The acquisition was performed with the FlexComp Infiniti™ wireless EEG system by Thought Technology Ltd.

The EEG was recorded with Ag-Ag/Cl electrodes from 3 channels over the motor cortex and close to the brain area responsible to generate foot movements. These channels were C3, C4, and CZ (according to the international 10/20 system of EEG electrode positions). These channels were referenced to both ears.

### 2.2. Experimental Paradigm

The experimental task was to imagine either forward or backward movement depending on a written command. 10 naïve subjects (mean age 25.6 ± 3.92), 9 males and 1 female, participated in this experiment. They all signed a consent and received a compensation of 25$ each session.

The experiment included two sessions for one and a half hour each, where data of session 1 were used in session 2 for online neurofeedback and for offline analysis.

Session 2 was performed in 2 days to avoid fatigue and to allow learning. Each session contained 4 runs, and each run was 80 trials (Table 1), which resulted in 320 trials in total, 160 trials for moving backward and 160 trials for moving forward. The number of trials was based on the Graz BCI paradigm, and to obtain enough trials for the averaging step. The subject stood in the Icube 50 cm away from the front screen, where then we started the built-up 3D environment.

**Figure 3:**
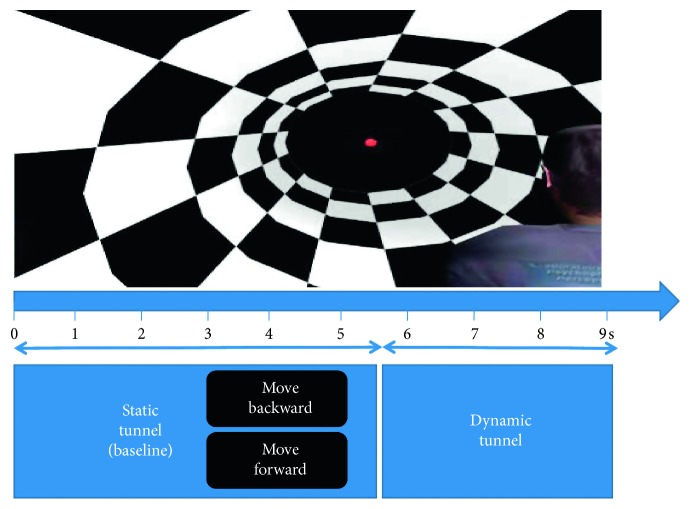
One trial paradigm.

Each trial was 9 seconds long and started with 3 seconds of a static tunnel, in order to acquire a baseline EEG, and then comes the presentation of a written command “Move Forward” or “Move Backward” randomly at the center of the screen for 2 seconds, followed by 0.5 seconds of a static tunnel and then 3.5 seconds of a dynamic tunnel (Figure 3).

The instructions the participants received were the following: If the written command was “Move Forward,” the subject had to imagine moving in the tunnel in a forward movement. However, if the written command was “Move Backward,” the subject had to imagine moving in the tunnel in a backward movement. The participant was instructed to stop the imagination once the tunnel started to move.

In session 1, the tunnel moved after the display of the written instruction, and at the same direction of this instruction, independently and regardless of the motor imagery results. However, in session 2, the tunnel moved as a neurofeedback of the participant's online real-time brain activity of motor imagery. The main part that distinguishes neurofeedback from the BCI is the BCI machine learning phase.

**Figure 4:**
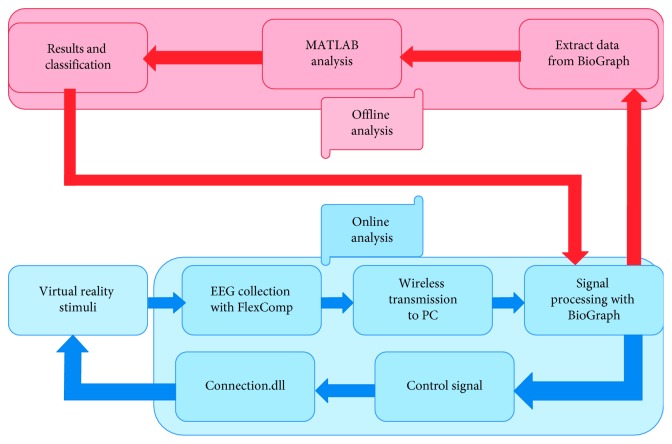
The overall system flow chart.

BioGraph (the software provided by the EEG device manufacturer Thought Technology Ltd.) was used for data acquisition and display and was synchronized with the virtual reality computers (Figure 4).

### 2.3. Offline Signal Processing

**Figure 5:**
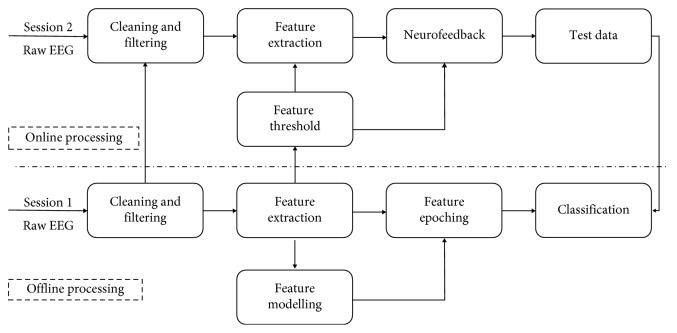
Signal processing flowchart. The bottom panel shows different stages of the offline signal processing for session 1, and the upper panel shows different stages of the offline signal processing for session 2.

After running subjects in session 1, data were processed offline, as depicted in Figure 5. The results were then used for the following:

1. To perform session 2 with online real-time neurofeedback
2. To build a classifier that used session 2 data as test data in an offline BCI simulation

#### 2.3.1. Offline Signal Processing: EEG Cleaning and Filtering

The EEG signals were amplified and bandpass filtered (18th order Butterworth IIR filter) between 8 and 30 Hz and sampled at 256 Hz and then bandpass filtered into four frequency bands: 8–12 Hz (alpha), 12–15 Hz (SMR), 16–24 Hz (beta), and 25–30 Hz (high beta).

Artifacts were autorejected within BioGraph, by automatically removing the parts of the recorded signal where the amplitude exceeds 20 *μ*v. Signals were then detrended, and clean data were used for offline analysis.

#### 2.3.2. Offline Signal Processing: Feature Extraction

*(1) Time-Frequency Representation (TFR)*. Performing a short-time Fourier transform (STFT) permits an insight into the ERD/ERS by analyzing the power variation with respect to time [29]. The STFT was estimated at a high spectrogram resolution of 1-second Hanning windows in time with a 50% overlap and 64 frequency bins.

The TFR (time-frequency representation) was mainly used for both online and offline visualization to define the most active frequencies and their strengths during the mental task achieved by the participant in every single trial.

*(2) Power Spectral Density (PSD) Features*. They inform on the distribution of the power of a signal between different frequencies [2]. A 1-second Hanning window was chosen to give a frequency resolution of 1 Hertz/bin within the periodogram, which computes PSD by squaring the signal Fourier transform.

*(3) Power Asymmetrical Ratio*. This method is especially useful in tasks that involve interhemispheric differences, where the power asymmetrical ratio is defined as

(1) $$\begin{array}{c} Ra=\frac{R-L}{R+L}, \end{array}$$

where *R* is the PSD or power of a specific band in the right electrode and *L* is the PSD or power of a specific band in the left electrode [2].

*(4) Band-Power (ERD/ERS) Calculation*. For EEG data, bandpass filtering of each trial, squaring of samples, and averaging of *N* trials result in a time course of instantaneous band power [30].

Then, the ERD is quantified as the percentage change of the power at each sample point or an average of some samples relative to the average power in a reference interval:

(2) $$\begin{array}{c} ERD{\%}_{\left(j\right)}=\frac{A_{\left(j\right)}-R}{R}\;\times \;100\%, \end{array}$$

where *R* = average power in the reference interval, averaged over *k* samples, and *A*_(*j*)_ = power at the *j*-th sample [10]. The best two ERS/ERD values that distinguished between two tasks can then be selected.

#### 2.3.3. Offline Signal Processing: Feature Modeling

*(1) Autoregressive (AR) Parametric Models*. In this study, we used the autoregressive (AR) Burg method and its extension ARX [31], which are generally used as features for the BCI to distinguish one time series from another [4]. For ARX model order selection, the Akaike information criterion (AIC) was applied in this study [32]. In this method, the input was assumed to have Gaussian statistics.

We set the exogenous input to be the template of averaged backward trials when modeling backward trials and the template of averaged forward trials when modeling forward trials because averaging across trials assists in extracting the event-related potential hidden within the noise.

#### 2.3.4. Offline Signal Processing: Feature Epoching

The previously mentioned extracted features and their models were divided into 4 epochs, in order to investigate the optimum time slot (and thus feature points) that the imagery signal achieved the best classification accuracy [33], thus decreasing the computation time. Various epochs were experimented based on a 0.5 s shift. These epochs were as follows:

1. Epoch 1: 3.5–4.5 s
2. Epoch 2: 4–5 s
3. Epoch 3: 4.5–5.5 s
4. Epoch 4: 5–6 s

#### 2.3.5. Offline Signal Processing: Classification Models

The previously mentioned extracted features and their AR models and epochs were used to train the following classifiers:

*(1) Linear Discriminant Analysis (LDA)*. The aim of LDA is to find a linear combination of features which separates two or more classes with *k*, *n*-space hyperplanes [2, 34]. In this study, we used the diagonal LDA, which computes the diagonal covariance matrix estimates.

*(2) Support Vector Machine (SVM)*. In this study, we used a linear SVM algorithm. See [2, 7, 35] for further details.

*(3) Classification Validation*. A 10-fold cross-validation was used. This algorithm breaks data into 10 sets of size *n*/10, trains on 9 datasets, and tests on 1 dataset; it repeats this process 10 times and finally takes a mean accuracy.

## 3. Results

### 3.1. Session 1: Visualization of TFR

**Figure 6:**
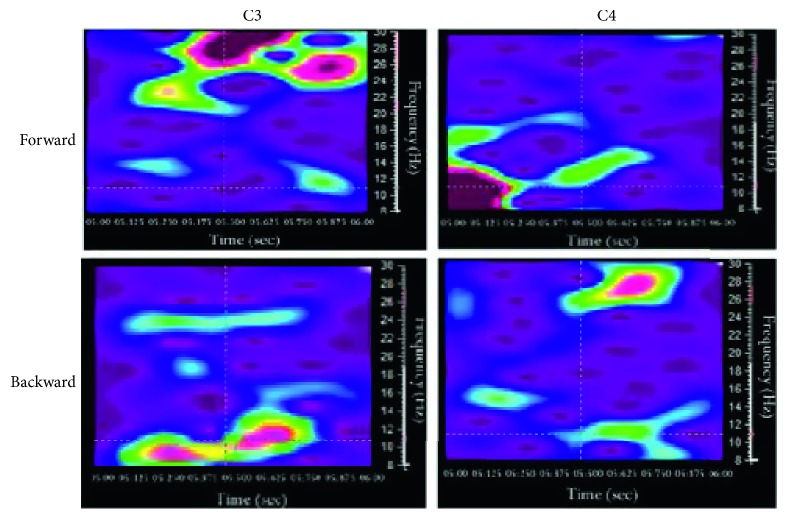
TFR over C3 and C4 for subject 3.

Figure 6 displays four online TFRs for subject 3. Almost the same patterns were generated by the rest of the participants. These TFRs were calculated within epoch 4, where the first half second of the TFR represents half a second before MI termination and the second half second of the TFR represents half a second after the MI termination.

The first column displays TFR for electrode C3, and the second column displays the TFR for electrode C4.

The first row refers to the TFR when the subjects were instructed to imagine a forward movement, and the second row refers to the TFR when the subjects were instructed to imagine a backward movement.

During the forward movement, we can clearly see the *α*-ERD, but almost at the end of the MI, a large and remarkably strong *β* ERS appeared over C3 and lasted for few milliseconds. On the contrary, the *α*-ERD appeared also during the backward movement over C3, however, and right after the end of the backward MI, a smaller yet strong *β* ERS appeared over C4 and lasted for few milliseconds.

This suggested that we had a lateralization of signals for MI forward and backward movements, where the right motor cortex showed higher activity for backward movement versus the left motor cortex, which in turn showed a higher activity for the forward movement.

This result supports our hypothesis that MI of forward-backward movement can activate the motor cortex the same way as a strong optic flow does [27].

**Figure 7:**
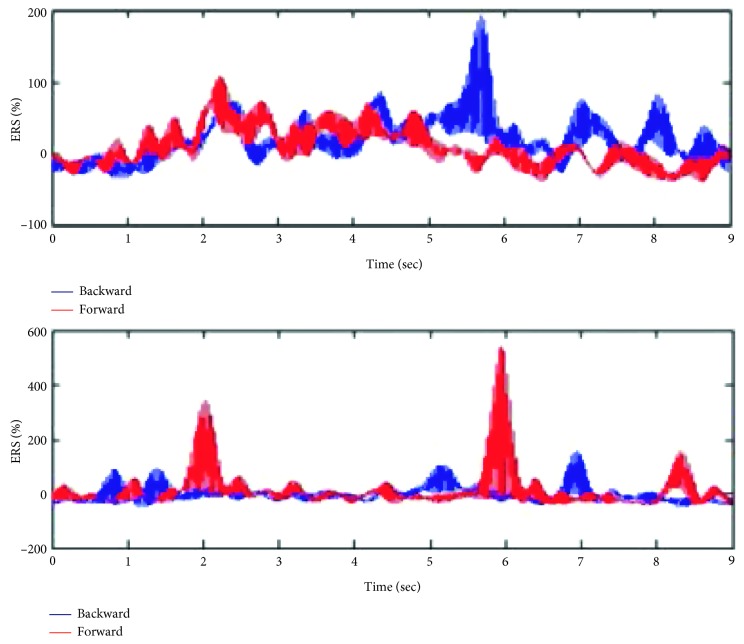
Grand average *β*-ERS at channels C4 (upper) and C3 (bottom).

Figure 7 presents the *β*-ERS at channels C4 and C3, respectively, and at the frequency band 25–30 Hz.

### 3.2. Session 1: Offline Calculation of Band-Power ERD/ERS

It shows a remarkable backward ERS that starts right after the termination of the movement imagery and peaked with 200% after about 200 msec. The template illustrates no overlap with the forward signal which appears to be equal to baseline, with a correlation of 0.31.

It also shows a remarkable forward ERS that starts 200 msec after the movement offset and right after the termination of the movement imagery and peaked with 600% after about 300 msec, thus 500 msec after the termination of the MI. The template illustrates no overlap with the backward signal which appears to be equal to baseline, with a correlation of 0.28.

This lateralization (shown in [27, 36]) states that MI of moving backward would relatively highly activate the motor cortex of the right brain hemisphere, resulting in a higher right ERS, while MI of moving forward would activate the motor cortex of the left brain hemisphere, resulting in a higher left ERS.

EEG channels C3 and C4 lie over the primary motor cortex, which is mainly responsible for movement planning and represents the hand movement area in the brain.

However, the foot motor area representation is located deep within the interhemispheric fissure, right under the EEG channel Cz that lies over the cranial midline; thus, the detection of ERD/ERS through the EEG signal is very difficult. This explains the reasons for the following:

1. Very small ERD power changes over Cz were detected
2. Many studies detected the brain activity of navigation only in EEG channels close to the channel Cz, such as Fz [37] and Pz [38]

On the contrary, the phenomena of ERD/ERS reflect the dynamics of neural networks and can be observed on different scalp locations at the same moment in time, whereas one cortical area displays ERD and other areas display ERS.

This phenomenon is called “focal ERD/surround ERS” and is interpreted as a “*correlate of an activated cortical area (ERD) and simultaneously deactivated or inhibited areas, very likely mediated by thalamic gating*” [10].

Thus, in this study, the ERS patterns that were found over hand representation areas were developed simultaneously with ERD in the area of the feet [39], which is also consistent with what studies [36, 40, 41] found.

### 3.3. Session 2: Online Real-Time Band-Power ERD/ERS Neurofeedback

The two ERS values that were obtained in session 1 (see Section 3.2) were set up in BioGraph as signal thresholds to form a control signal for an online real-time neurofeedback of the real-time processed data in session 2.

The first ERS value was for transition from the idling to the “move forward” state, while the second one was for transition from the idling to the “move backward” state. So, the backward threshold was set on the BP of the frequency band 25–30 Hz over channel C4 according to the following equation:

(3) $$\begin{array}{c} {\text{Th}}_{b}=\text{MEAN}+6\times \text{STDEV,} \end{array}$$

where Th is the threshold, *b* refers to backward, MEAN is the power calculated in a reference interval, and STDEV is the standard deviation.

The STDEV coefficient was calculated according to the ERS time course and was selected based on a value that would yield a maximal separation between the standard deviations from the mean baseline of the two tasks and was set to 3 standard deviations each, i.e., 3 negative standard deviations from the mean of the task that had the higher curve and 3 positive standard deviations from the mean of the task that had the lower curve.

For subject 7, we calculated two negative standard deviations from the mean of the averaged backward signal over C4 and calculated its ERS time course and found that it was close to 3 standard deviations from the mean baseline. We then repeated that for the forward signal and found that it was 2 standard deviations from the mean baseline. So we selected the value 6 based on 2 STDEV_backward_ + 1 STDEV_safety for backward_ + 3 STDEV_forward_.

This formula means that whenever the power over the motor cortex of the right hemisphere exceeds and passes a threshold of 6 standard deviations from the baseline, i.e., activation over C4 resulting from the MI of backward movement, this would trigger the virtual reality tunnel with a boolean that induces the tunnel to move in the backward direction. Similarly, the same setting was used as a threshold over the motor cortex of the left brain hemisphere.

Session 2 consisted of four runs, performed in 2 different days, of the same week, where the same paradigm was used but with two differences.

The first difference was that, in session 2, the subject was instructed to stop the MI right after the command “Move Forward” or “Move Backward” offset; that is, at second 5 of the trial, instead of stopping the MI once, the tunnel moves.

This modification was based on the ERS results. Since the ERS peaked at 200–500 ms of the MI offset for both tasks, this modification of terminating the MI at second 5 would let the beta power to rebound and then peak at second 5.5.

The Interface software between BioGraph and our virtual tunnel was programmed such that whenever the signal passed the threshold at second 5.5 and only at this point, the boolean was sent to trigger the tunnel to move in either direction.

In session 2, EEG was downsampled to 8 Hz and smoothed out by averaging 3 consecutive samples (with a moving average) in order to produce a smooth control signal (CS).

Amongst the 10 subjects we tested, 7 showed the same ERS pattern at the high *β* frequency band, and 3 subjects showed the same pattern but with a central *β*-ERS (16–24 Hz). All patterns had also different ERS values for both forward and backward MI.

**Figure 8:**
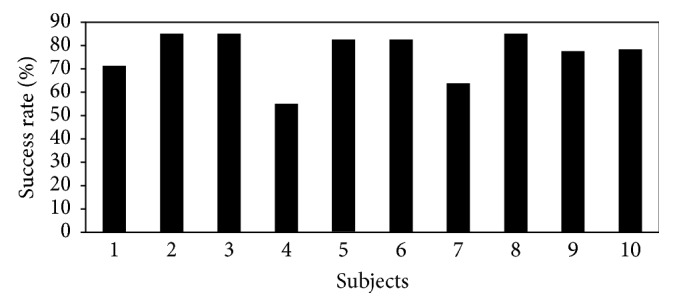
Run 4: average accuracy for all subjects.

Figure 8 shows that 50% of the subjects were able to achieve accuracy more than 80% and after a total of 96 minutes of training.

**Figure 9:**
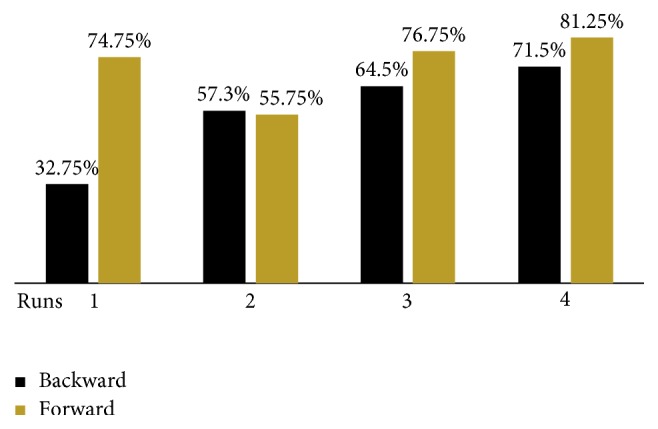
Beta-ERS method accuracy across runs and averaged across all subjects.

Figure 9 shows success rates across runs and averaged across subjects. We can see an expected bias toward forward control versus backward control in run 1. We have also applied the *t*-test to interpret the results, where we applied the intrasubject *t*-test on the pair of the two tasks in every single run.

The subjects had significantly more trouble in controlling the tunnel movement using backward MI more than the forward MI (*r*_b1-f1_ = 0.191, *t* = −7.434, dof = 9, *p* < 0.001) because walking forward is an easy automated task for the brain; however, moving backward would require more strength and concentration and would also require learning to develop a special mental strategy in order to alter the brain waves to produce the correct control signal.

So, the success rate for the backward control was 32.75% ± 17.53 versus 74.75% ± 8.11 for forward control. Most of the subjects in run 2 put much effort into the backward-control strategy learning versus the forward control, where the bias was inverted toward the backward control, in which its success rates were improved, accompanying a drop in the success rate of the forward control, with an average success rate of 57.3% ± 7.91 for backward control versus 55.75% ± 12.25 for forward control (*r*_b2-f2_ = 414, *t* = 0.426, *p* < 1).

The next day and in run 3, the backward control continued to improve gradually in an almost linear learning curve, where the forward control was regained (*r*_b3-f3_ = −0.181, *t* = −1.663, *p* < 0.2).

We think that was because this run was performed in a different day, and so the brain was back to achieve a high success rate for its automated task, the forward control, but sustained the learning and mental strategy developed for the backward control, and continued to develop it more, to achieve a higher success rate of 64.5% ± 16.02, where the forward control was regained with a success rate of 76.75% ± 14.24.

In run 4, the subjects' brains continued to develop the mental strategy for the backward control, where a remarkable improvement took place to increase the success rate with only 71.5% ± 13.44, where the forward control stabilized with slight improvement (*r*_b4-f4_ = 603, *t* = −2.864, *p* < 0.02).

So we can say that the neurofeedback induced a learning curve to control the backward navigation in virtual reality (*r*_b1-b4_ = −0.184, *t*_b1-b4_ = −5.109, *p* < 0.05), in contrast to the forward navigation in virtual reality (*t*_f1-f4_ = −1.816, *p* > 0.1), where in this neurofeedback, the learning curve started with a bias toward the naturally automated forward control versus backward control and then the backward control improved gradually with a so-like linear learning curve; however, the forward control was lost in run 2 but regained and then stabilized at run 3 and 4.

We have applied ANOVA to the results for statistical comparisons. As a consequence of the numerous planned comparisons required in the first phase of this study, the Greenhouse–Geisser and Huynh–Feldt corrections were applied to interaction tests in order to control for random outcomes in this context.

The ANOVA revealed a significant intrasubject main effect of “Task” (*F* = 26.085, dof = 1, *p* > 0.01), a significant effect of “Runs” (*F* = 17.204, dof = 3, *p* < 0.001), and a significant “Runs” × “Task” interaction (*F* = 14.085, dof = 3, *p* < 0.01) and also revealed a significant intersubject main effect (*F* = 978.355, dof = 1, *p* < 0.001).

### 3.4. Session 1: Offline Classifications

The features extracted were frequency bands × epochs × sampling frequency × features × feature models' orders × channel combinations × subjects. Then, these features were used to train LDA and SVM classifiers, where all these classifications were performed with 10-fold cross-validation. The offline nature of this analysis allowed us to run the tests on all of these features and then to select the classifiers/models that achieved the best classification performance.

**Figure 10:**
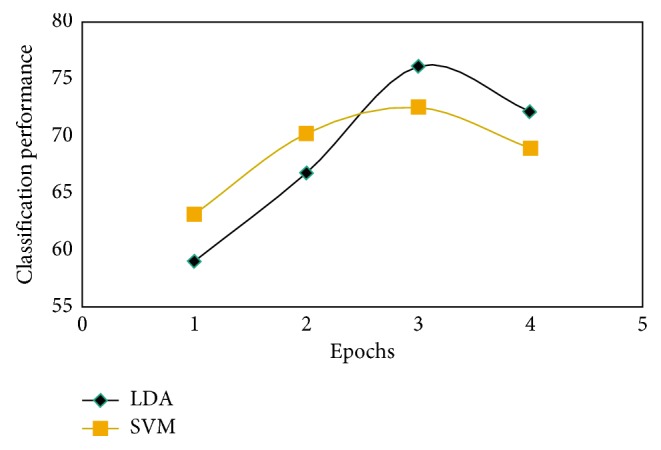
Classification of PSD-Ar between C3 and C4 within the *β* band varying with epochs and averaged across 10 subjects.

To investigate the best classification in terms of epochs, classification was run over the 4 epochs. The results showed that the epoch that achieved the highest classification accuracy was epoch 3 (Figure 10), i.e., the time slot that started 1.5 seconds after the command display and ended just before the tunnel started to move.

This means that the classifier gave poorer results when classifying the ERD and better results when classifying the ERS. However, the classifiers' performance was attenuated at epoch 4; we think that this was due to the visual effect the dynamic tunnel added to the ERD/ERS. All coming results are presented within epoch 3.

To investigate the best classification in terms of channels and rhythms, classification was run over single and then different combinations of channels and over alpha, SMR, and beta rhythms. Results show poor prediction within alpha and SMR and poor prediction when using the single EEG channel, so we cannot rely on one single EEG channel to predict the upcoming forward-backward movement and we need at least two EEG channels to achieve an average accuracy more than 75% when predicting the upcoming forward-backward movement within the beta band.

So, all results that will be presented next are within the beta band and between C3 and C4 EEG channels.

To investigate the best features, we ran classification over BP and PSD-Ar and over their AR and ARX models, varying the model order from 2 to 30, with a step value of 1.

To investigate the best classification in terms of classifiers (algorithms), we used linear LDA and linear SVM. For SVM, we tested different values for the *C* factor in an exhausted search for the optimum *C* factor, where we varied the values exponentially between 1·*e*^−7^ and 1·*e*^2^ with a step value of e^−1^.

However, we ran the search for the optimum *C* factor on the data of randomly selected 50% of the subjects, which means we tested 5 subjects out of the 10 subjects and found *C* = 1·*e*^−2^ to give optimum results, so we assumed a generalization of this value for all subjects' data in this study. All results that will be presented next are presented with the *C* factor that achieved the highest accuracy, *C* = 1·*e*^−02^.

The LDA classifier gave a 6% higher accuracy than the SVM, but the classification drops remarkably when the features were modeled in both AR and ARX methods, even when these models were modeling noise but not the actual feature; thus, they were poor at predicting upcoming forward and backward movements. Thus, using the PSD asymmetrical ratio as a feature gave better results than using their model coefficients as features.

Table 2 shows comparison between different session 1 offline classification accuracies over PSD-Ar features when fed to LDA, using 2 EEG channels, and over band-power features when fed to SVM, using 3 EEG channels, and using the best AR and ARX model orders, for all subjects, and then averaging over all subjects.

So, using the 2-channel BCI, classification models' performance over session 1 data showed that only 20% of the subjects achieved a classification accuracy of 80–82% and that 60% achieved a classification accuracy of 70–80%.

Statistical tests were run to investigate if there were significant differences between these classification models and then to select the classification models that achieved the best classification performance.

The Shapiro–Wilk test revealed that the normality assumption is rejected, so the Wilcoxon test was performed over all paired samples of the models' classification accuracies. This statistical test revealed many important findings.

First, no significant differences were found between using the PSD-Ar features and using their filtered autoregressive models when feeding them to either LDA (*Z* = 1.3239, *p* > 0.1, and *Z* = 1.0220, *p* > 0.3) or SVM (*Z* = 0.6050, *p* > 0.5, and *Z* = − 0.2646, *p* > 0.7) classifier nor between using any of the two autoregressive models used in this study (*Z* = −0.7181, *p* > 0.4).

Second, the statistical tests revealed significant differences when using all LDA models versus all SVM models (*p* < 0.05).

### 3.5. Session 2: Offline Classifications

Section 3.4 showed that the two classification models of session 1 that achieved the best performance and were significantly different were the following:

1. Classifier 1: *β*-PSD-Ar between C3 and C4 with LDA
2. Classifier 2: *β*-band power modeled with AR_burg_ over C3, C4, and Cz with the SVM classifier

These 2 trained classification models were used offline over processed data from session 2, in a so-like BCI simulation of the classifier performance if implemented online.

Results in Table 3 show that classification model 2 reached an average accuracy over all subjects of 80%.

The performance of model 2 used for offline classification was 4% higher than the performance of the model used for online neurofeedback. The statistical tests confirmed the nonsignificant differences between these 3 models (*p* > 0.1*p*).

## 4. Conclusion

This study investigated, in a first session, the brain activity of the MI of forward/backward navigation in a virtual tunnel, where many models from the data of this session were then trained. Then, in a second session, only one model was used online in real time, which is the band-power neurofeedback, since the remaining models were machine learning models, which can be tested offline over the data of this second session.

Supporting previous psychophysical studies, this study showed that the motor cortex was activated bilaterally when backward-forward feet movements were imagined, and thus, two EEG channels must be used to control a VR application via a BCI using MI of lower limbs' backward and forward movements. To our knowledge, this is the first study to investigate a BCI that used the MI moving backward command. It is also the first to compare the use of forward and backward MI commands to activate a BCI and to compare the performance of neurofeedback and the performance of a BCI simulation that uses different algorithms, in order to control the navigation in a VR environment using MI of lower limbs' backward and forward movements.

All subjects were able to control their navigational direction within the tunnel, but with an averaged accuracy of 76%. The subjects found the backward control a harder task to achieve (the average accuracy for backward control was 50–70% versus ∼64–84% for forward control).

The band-power features modeled with AR and classified with SVM as well as the well-known and widely used power spectral asymmetrical ratio with the LDA classifier gave slight better results than human learning methods (*p* > 0.1), with an average classification accuracy of almost 80%. Using autoregression modeling over the band-power features helped in decreasing the number of features over one channel but increased the required number of channels, where good classification accuracy was obtained only when using band-power features from three channels.

For the future work, and since MI of backward movement required higher strength to achieve and control, another strategy to improve the BCI control is to assign an easier imagery task to control the backward navigation in the tunnel. Also, more machine learning algorithms will be investigated, in order to improve the performance and capabilities to control this BCI.

## Figures and Tables

**Table 1:** Methodology and protocol.

1 session = 4 runs	
1 run = 80 trials	
1 run = 12 min	10 min pause

**Table 2:** A comparison between session 1 offline classification accuracies over PSD-Ar and BP features using different methods and across all subjects.

Subject	LDA	SVM	AR-LDA	AR-SVM	ARX-LDA	ARX-SVM
1	74.5	68.57	72.79	69.44	73.38	73.71
2	76.84	71.85	77.63	70.84	75.29	67.84
3	80.44	78.88	78.88	75.82	78.88	76.5
4	73.38	67.75	74.91	66.5	73.38	72.13
5	69.79	65.35	64.5	65	68.88	66.69
6	69.5	67.16	68.25	69.91	71.47	63.38
7	79.82	71.07	73.38	60.75	77.52	70.69
8	77.54	72.47	74.5	75.57	75.6	77.57
9	81.54	71.69	75.29	71.69	76.5	70.07
10	77.79	67.32	74.35	66.85	76.85	68.44
AV	76.11	70.21	73.45	69.24	74.77	71.2
STDEV	4.23	3.89	4.24	4.66	3.02	3.72

**Table 3:** A comparison between the real-time online neurofeedback performance and the offline best classifier performance over session 2.

Subject	PSD-AsR-LDA	BP-AR-SVM	Neurofeedback
1	78.3	79.34	71.25
2	83.25	83.43	85
3	78.74	84.67	83.75
4	78.5	79.06	55
5	75.5	82.81	82.5
6	73.84	75.31	82.5
7	79.44	79.86	63.75
8	80.22	84.06	85
9	80.63	74.68	77.5
10	81.02	75.93	77.5
AV	78.94	79.915	76.38
STDEV	2.71	3.74	10.13

## Data Availability

The data used to support the findings of this study are available from the corresponding author upon request.
